# Supplementary material for: Alpha-Lipoic Acid Ameliorates Radiation-Induced Salivary Gland Injury by Preserving Parasympathetic Innervation in Rats
Source: Int J Mol Sci. 2020 Mar 25;21(7):2260. doi: 10.3390/ijms21072260 (PMC7178006; doi:10.3390/ijms21072260)
Supplement: Supplementary file 1 [file ijms-21-02260-s001.pdf]

Supplementary:

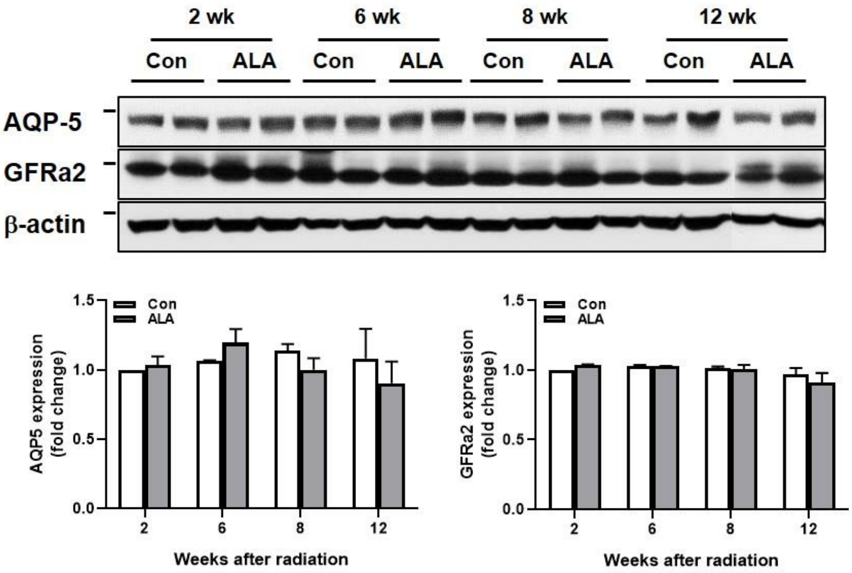


**Supplementary Figure 1.** Salivary gland expression of AQP5 and GFRa2 in 2, 6, 8, and 12 weeks after radiation. β-actin was used as the loading control. Western blots for the expression of AQP5 and GFRa2 are normalized to β-actin and the expression was shown as signal intensity. Figures are representative in each group. Size markers mean 37, 50, and 50 kDa from upper. Con; control (*n* = 3 per lane). ALA; ALA only treated group (*n* = 3 per lane). Data were presented as mean±SEM ($*p <$ 0.05).
